# Knowledge and practices on childhood anaemia, thalassaemia and iron deficiency among mothers of children aged between 6 and 59 months in a suburban area of Sri Lanka

**DOI:** 10.1186/s41043-022-00341-7

**Published:** 2022-12-31

**Authors:** Ruwan Samararathna, A. V. C. Gunaratne, Sachith Mettananda

**Affiliations:** 1 Colombo North Teaching Hospital, Ragama, Sri Lanka; 2 Department of Paediatrics, Faculty of Medicine, University of Kelaniya, Thalagolla Road, Ragama, 11010 Sri Lanka

**Keywords:** Anaemia, Iron deficiency, Thalassaemia, Nutritional anaemia, Haemoglobinopathy

## Abstract

**Background:**

Childhood anaemia is one of the most common public health problems worldwide. Here, we aim to describe the knowledge and practices on childhood anaemia, thalassaemia and iron deficiency among mothers of children aged between 6 and 59 months in a suburban district of Sri Lanka.

**Methods:**

We performed a cross-sectional survey in the Gampaha District of Sri Lanka from December 2020 to February 2021. One well-baby clinic each from four Medical Officer of Health areas in the district was selected using stratified random sampling. Mothers of all children aged between 6 and 59 months attending well-baby clinics were recruited until the sample size was achieved. Data were collected using a self-administered questionnaire and analysed using logistic regression.

**Results:**

A total of 392 mothers were recruited; 53% of their children were males. Only 33% of mothers had an accurate understanding of anaemia, while 71% and 28%, respectively, could name at least one symptom and two causes of anaemia; 12% could not name a single food rich in iron. Only 13% of mothers knew that thalassaemia is a cause of anaemia, and 14% had been screened for thalassaemia. Logistic regression analysis that examined for factors associated with higher knowledge of anaemia revealed that an accurate understanding of anaemia was associated with maternal age over 30 years (*p* < 0.05) and maternal education level beyond grade ten (*p* < 0.001). In contrast, higher knowledge of symptoms of anaemia was associated with maternal employment (*p* < 0.01).

**Conclusions:**

The knowledge of anaemia and awareness of thalassaemia among mothers was poor. Very few mothers were aware of iron-rich food and feed it to their children. Despite being located in a thalassaemia-endemic region, very few knew that thalassaemia is a cause of anaemia and have got themselves screened for thalassaemia.

**Supplementary Information:**

The online version contains supplementary material available at 10.1186/s41043-022-00341-7.

## Background

Anaemia is one of the most common public health problems in the world [1]. It is estimated that one-third of the world’s population suffers from anaemia [2]. Anaemia is most common in the low-and-middle-income countries in sub-Saharan Africa and south and southeast Asia [3]. The prevalence of anaemia is higher in certain high-risk groups that include children, pregnant women and the elderly [4]. Children between 6 and 59 months are particularly at risk of developing anaemia compared to other times of life [5]. In Sri Lanka, the prevalence of anaemia among preschool children is reported as 15% [6].

Anaemia in children has several causes. Iron deficiency is the most common cause of anaemia among preschool children globally [7]. Iron is an important cofactor of the haemoglobin molecule; therefore, iron deficiency leads to anaemia. The prevalence of iron deficiency anaemia among children in Sri Lanka is reported as 7.3%, which is mostly caused by inadequate intake of dietary iron [1].

Heterozygous carrier states of haemoglobinopathies such as β-thalassaemia trait and α-thalassaemia trait are other common causes of anaemia in children [8, 9]. Thalassaemia is caused by mutations in the α- and β-globin genes that encode for α- and β-globin polypeptide chains of the haemoglobin molecule [10]. Thalassaemia trait is an asymptomatic condition which results in mild microcytic anaemia [11]. It does not require any treatment; however, the affected individuals should avoid marrying a partner with thalassaemia trait to prevent the birth of a child with severe homozygous forms of thalassaemia (thalassaemia major) [12]. The prevalence of β- and α-thalassaemia traits in Sri Lanka is reported as 2.8% and 8%, respectively [13, 14]. Most importantly, thalassaemia trait and iron deficiency coexist among Sri Lankan children [15].

Sri Lanka has a state-run National Thalassaemia Prevention Programme functioning for the past 15 years [16]. The strategy used by this programme is to advocate voluntary free screening of all young individuals to identify thalassaemia carrier states. Once identified, thalassaemia carriers are advised to avoid marrying another thalassaemia carrier and to have ‘safe marriages’ with partners who are free from thalassaemia. Thereby, the programme expects to prevent the births of children with thalassaemia major and gradually bring down the incidence of thalassaemia [17].

One strategy for preventing iron deficiency and anaemia in children is increasing public awareness of the condition [4]. In particular, parents of children in at-risk age groups should be well aware of anaemia and iron deficiency. However, the degree of awareness of parents of preschool children about the causes and features of anaemia has not been adequately studied in Sri Lanka. In this study, we aim to describe the knowledge and practices on childhood anaemia, thalassaemia and iron deficiency among mothers of children aged between 6 and 59 months in the Gampaha District of Sri Lanka.

## Methods

We conducted a cross-sectional survey in the Gampaha District of the Western province of Sri Lanka between December 2020 and February 2021. Gampaha District is located near the capital of Sri Lanka and is the most populated district of Sri Lanka. It is home to 11.3% of the Sri Lankan population, with a population density of 1700/km^2^. It has 16 Medical Officer of Health (MOH) areas.

Mothers of children aged between 6 and 59 months attending well-baby clinics of the Gampaha District during the study period were eligible to participate in this study. Mothers of children with transfusion-dependent thalassaemia and neurological or feeding difficulties were excluded. Four out of 16 MOH areas of the Gampaha District were randomly selected for the study. Eligible mothers of all children aged between 6 and 59 months attending a randomly selected well-baby clinic of each MOH area during the study period were recruited into the study after obtaining informed written consent. The sample size was calculated using the standard formula used for descriptive and prevalence studies [*N* = *Z*^2^*P*(1 − *P*)/*d*^2^] for a confidence interval of 95% and a precision of 5% [18]. The calculated minimum sample size was 384.

Data were gathered using a self-administered pretested questionnaire prepared in all three languages. Initially, the English questionnaire was prepared and translated into Sinhala and Tamil by professional translators. Then, a third party back-translated the Sinhala and Tamil questionnaires into English to identify any disparities. The pilot study was carried out in the University Paediatrics Unit of Colombo North Teaching Hospital, Ragama, using ten parents of children aged between 6 and 59 months. No changes were required to the questionnaire or the data collection procedure after the pilot study. The study participants were given the questionnaire in the language they were most conversant with. All participants had the ability to read, comprehend and answer the questionnaire at least in one language. The completed questionnaires were collected on the same day.

Statistical analysis was done using IBM SPSS version 22.0. Categorical variables were presented as frequency and percentages. Binary logistic regression was used to determine associations between categorical variables, and adjusted odds ratios (AOR) were presented. The logistic regression models that examined the independent associations for higher knowledge of anaemia included mother’s age, mother’s education level, mother’s employment status, father’s occupation, monthly family income and number of children at home as covariates. These sociodemographic factors were selected because they were considered to be associated with parental knowledge of health-related issues in previous studies conducted in Sri Lanka [19–21]. The variables were transformed into dichotomised variables considering the local context for socially meaningful cut-offs and as used in previous studies [19–21]. The cut-off for statistical significance was set at *p* < 0.05.

Ethical clearance was obtained from the Ethics review committee of the Sri Lanka College of Paediatricians (Reference number: SLCP/ERC/2020/28). Administrative clearance was obtained from the Regional Director of Health Services of the Gampaha District and the selected MOH.

## Results

A total of 392 mothers were recruited into the study. A majority were aged between 26 and 35 years and were housewives. The sociodemographic characteristics of the study population are given in Table 1.

**Table 1:** Sociodemographic characteristics of the study population

Sociodemographic characteristic	Frequency (*N* = 392)	Percentage (%)
Sex of the child		
Male	210	53.6
Female	182	46.4
Mother’s age		
< 20 years	3	0.8
21–25 years	49	12.5
26–30 years	104	26.5
31–35 years	131	33.4
36–40 years	81	20.7
41–45 years	22	5.6
> 46 years	2	0.5
Mother’s education level		
No education	2	0.5
Primary	3	0.8
Up to GCE O/L	170	43.4
Up to GCE A/L	174	44.4
Higher education	43	11.0
Mother’s occupation		
Housewife (Full-time caretaker)	275	70.2
Unskilled	10	2.6
Skilled	41	10.5
Lower professional	58	14.8
Higher professional	8	2.0
Father’s age		
21–25 years	26	6.6
26–30 years	66	16.8
31–35 years	132	33.7
36–40 years	116	29.6
41–45 years	45	11.5
> 46 years	7	1.8
Father’s education level		
No education	0	0
Primary	6	1.5
Up to GCE O/L	211	53.8
Up to GCE A/L	144	36.7
Higher education	31	7.9
Father’s occupation		
Unemployed	0	0.0
Unskilled	45	11.5
Skilled	234	59.7
Lower professional	91	23.2
Higher professional	22	5.6
Monthly family income		
≤ LKR 25,000	49	12.5
LKR 25,001–50,000	221	56.4
LKR 50,001–100,000	83	21.2
> LKR 100,000	39	9.9
Number of children in the family		
One	156	39.8
Two	160	40.8
Three	65	16.6
Four	8	2.0
Five or more	3	0.8

### Maternal knowledge of symptoms and causes of anaemia

An accurate understanding of the term anaemia as either a reduction in haemoglobin or a reduction in red blood cells was known by only 131 (33.4%) mothers (Table 2). Most (30.2%) of them thought the reduction in body iron is known as anaemia. Approximately one-fourth (24.7%) had no idea about anaemia. More than 25% of the mothers could identify at least four symptoms of anaemia. Iron deficiency was identified as a cause of anaemia by 326 (83.2%) mothers. However, vitamin B12 deficiency, folic acid deficiency, thalassaemia and other aetiologies were known by less than 15%.

**Table 2:** Knowledge of anaemia and iron deficiency

	Frequency (*N* = 392)	Percentage (%)
What do you understand by anaemia?		
Reduction in haemoglobin	87	22.2
Reduction in red blood cells	44	11.2
Reduction in body iron	117	30.2
Reduction in blood volume	47	12.0
Not known	97	24.7
Accurate identification of symptoms of anaemia		
Irritability	42	10.7
Loss of appetite	108	27.6
Fatigue	158	40.3
Faintness	109	27.8
Dyspnoea	38	9.7
Pica	53	13.5
Developmental delay	172	43.9
Accurate identification of causes of anaemia		
Iron deficiency	326	83.2
Folate deficiency	46	11.7
Vitamin B12 deficiency	52	13.3
Hookworm infestations	35	8.9
Thalassaemia	54	13.8
How many iron-rich foods could the mother correctly name		
None	49	12.5
One	26	6.6
Two	53	13.5
Three	69	17.6
Four	62	15.8
Five	133	33.9
Knowledge of inhibitors of iron absorption		
Breastfeeding after a meal reduces iron absorption	34	8.7
Tea after a meal reduces iron absorption	253	64.5

### Knowledge of iron deficiency and factors associated with poor iron absorption

Out of all participants, 133 (33.9%) mothers could name five food rich in iron. However, 49 (12.5%) could not name a single iron-rich food (Table 2). A majority stated that lemon has a high amount of iron. Two hundred fifty-three (64.5%) mothers knew that drinking tea after a meal can reduce iron absorption, but only 34 (8.7%) knew that feeding milk after a meal also has a similar effect.

### Practices of giving iron-rich food

Analysis of data revealed that only about 9 (2.3%), 56 (14.3%) and 69 (17.6%), respectively, add meat, fish and egg daily to their child’s meals. However, 38 (9.7%) had not given meat to their children even once. A majority of mothers (360, 91.8%) were able to give an egg at least once a week, and 315 (80.3%) children received meat at least once a week. Fifty-six (16.4%) received a daily portion of fish, whereas 365 (93.1%) of the mothers gave fish with a main meal at least once a week (Table 3).

**Table 3:** Consumption of iron-rich food

	Meat	Fish	Egg
Daily	9 (2.3%)	56 (14.3%)	69 (17.6%)
2–3 per week	173 (44.1%)	237 (60.5%)	203 (51.8%)
Once a week	133 (33.9%)	72 (18.4%)	88 (22.4%)
Less than once a week	39 (9.9%)	25 (6.4%)	15 (3.8%)
Never	38 (9.7%)	2 (0.5%)	17 (4.3%)

### Practice of being screened for thalassaemia

Thalassaemia is an important contributory factor for anaemia in Sri Lanka. The National Thalassaemia Prevention Programme recommends that every person be screened for thalassaemia before marriage. However, in this study population, only 56 (14.3%) mothers have been screened for thalassaemia. Out of them, only 23 (5.9%) were screened before their marriage, whereas 16 (4.1%) were screened during pregnancy, and another 4 (1%) screened after childbirth.

### Factors associated with the accurate understanding of anaemia

We examined the factors associated with the accurate understanding of the term ‘anaemia’ as either a reduction in haemoglobin or a reduction in red blood cells (Table 4). An accurate understanding of anaemia was associated with maternal age over 30 years (AOR = 1.73, *p* < 0.05) and maternal education level beyond grade ten (AOR = 3.20, *p* < 0.001). Maternal employment status, father’s occupation, family income or the number of children in the family did not significantly correlate with an accurate understanding of the term anaemia.

**Table 4:** Factors associated with higher knowledge of anaemia

Sociodemographic factor	Has an accurate understanding of the term anaemia			Has the ability to name at least one symptom of anaemia			Has the ability to name at least two causes of anaemia		
	Number (%)	AOR (95% CI)	*p* value	Number (%)	AOR (95% CI)	*p* value	Number (%)	AOR (95% CI)	*p* value
Mother’s age									
> 30 (*N* = 236)	98 (41.5%)	1.73 (1.03–2.89)	< 0.05	178 (75.4%)	1.27 (0.78–2.06)	0.32	81 (34.3%)	1.32 (0.78–2.22)	0.29
≤ 30 (*N* = 156)	33 (21.2%)			100 (64.1%)			32 (20.5%)		
Mother’s education level									
> GCE O/L (*N* = 217)	101 (46.5%)	3.20 (1.90–5.40)	< 0.001	164 (75.6%)	1.12 (0.69–1.83)	0.63	73 (33.6%)	1.18 (0.70–1.99)	0.52
≤ GCE O/L (*N* = 175)	30 (17.1%)			114 (65.1%)			40 (22.9%)		
Mother’s employment status									
Employed (*N* = 117)	56 (47.9%)	1.55 (0.92–2.61)	0.09	99 (84.6%)	2.39 (1.31–4.37)	< 0.01	50 (42.7%)	2.21 (1.30–3.74)	< 0.01
Housewife / Full-time caretaker (*N* = 275)	75 (27.3%)			179 (65.1%)			63 (22.9%)		
Father’s occupation									
Professional (*N* = 113)	49 (43.4%)	1.14 (0.67–1.92)	0.61	87 (77.0%)	1.07 (0.61–1.87)	0.79	44 (38.9%)	1.43 (0.85–2.42)	0.17
Non-professional (*N* = 279)	82 (29.4%)			191 (68.5%)			69 (24.7%)		
Monthly family income									
> LKR 50,000 (*N* = 122)	57 (46.7%)	1.23 (0.72–2.11)	0.44	100 (82.0%)	1.64 (0.90–2.99)	0.10	47 (38.5%)	1.17 (0.67–2.04)	0.57
≤ LKR 50,000 (*N* = 270)	74 (27.4%)			178 (65.9%)			66 (24.4%)		
Number of children at home									
> 1 Child (*N* = 236)	89 (37.7%)	1.57 (0.95–2.58)	0.07	176 (74.6%)	1.53 (0.94–2.46)	0.08	83 (35.2%)	2.23 (1.33–3.75)	< 0.01
1 Child (*N* = 156)	42 (26.9%)			102 (65.4%)			30 (19.2%)		

The analysis was done using binary logistic regression. Adjusted odds ratios (AOR) were determined by adjusting to all other variables in the table

### Factors associated with the knowledge of common symptoms of anaemia

We then looked at the factors associated with a knowledge of symptoms of anaemia (Table 4). A significantly higher proportion of mothers who were employed could name at least one symptom of anaemia compared to non-working mothers (AOR = 2.39, *p* < 0.01). Maternal age, education status, family income or the number of children in the family did not show a significant association with the knowledge of symptoms of anaemia.

### Factors associated with the knowledge of common causes of anaemia

We also evaluated the factors associated with the knowledge of the causes of anaemia (Table 4). This revealed that a significantly higher proportion of mothers who were employed could name at least two common causes of anaemia compared to non-working mothers (AOR = 2.21, *p* < 0.01). Also, mothers with more than one child at home had significantly better knowledge of the causes of anaemia compared to mothers who had a single child (AOR = 2.23, *p* < 0.01).

## Discussion

In this study, we report maternal knowledge and practices on childhood anaemia, thalassaemia and iron deficiency in a highly populated suburban district of Sri Lanka. We found that only one-third of mothers had an accurate understanding of anaemia and a large proportion thought anaemia was synonymous with iron deficiency. Also, over 20% of mothers did not know the meaning of anaemia.

Our findings were comparable to the figures reported from other low-and middle-income countries evaluating maternal knowledge of anaemia. A study from Indonesia reported that only 35%-36% of mothers had knowledge of anaemia measured as their ability to name at least one symptom of anaemia and a strategy for reducing anaemia [22]. In a study done among pregnant women in Tanzania, only 35% of the respondents could define anaemia [23]. In contrast, a study done in Ethiopia reported that 54% of women had good knowledge of anaemia [24].

Our study also found that maternal knowledge of the symptoms of anaemia was poor. Although approximately 40% identified fatigue as an important symptom, the majority did not know other features of anaemia. This was similar to a recent study done in the Philippines, which revealed that only about 45% of women knew about the signs and symptoms of anaemia [25].

Regarding the causes of anaemia, most mothers (83%) knew that iron deficiency is the most common cause of childhood anaemia. However, only 13% knew that thalassaemia is a cause of anaemia. Although this was better than the reports from Bangladesh, where only 3% of mothers of children with thalassaemia had heard about the disease before it was diagnosed in their children, the figures are very low [26]. This is alarming as haemoglobinopathies are the second most common cause of anaemia in Sri Lanka [27, 28]. This fact highlights the need for intense public education regarding the common causes of anaemia relevant to the country.

Another fact revealed in this study is that only 14% of mothers had screened themselves for thalassaemia, and only 6% had done that before their marriage. The National Thalassaemia Prevention Programme of Sri Lanka recommends all couples test themselves before marriage and, in fact, before selecting their marriage partner[29, 30]. The facilities for screening for thalassaemia are available throughout the country for free [31, 32]. The low figure reported in our study highlights that despite its existence for over 15 years, the National Thalassaemia Prevention Programme has not been able to deliver the message to the public [12]. In a recent study done among 245 females diagnosed with anaemia during pregnancy, we found that 18 had undiagnosed β-thalassaemia trait, which was not recognised up to the pregnancy [33]. Therefore, it is timely that we take measures to conduct a clinical audit to evaluate the effectiveness of the National Thalassaemia Prevention Programme in Sri Lanka.

Regarding the knowledge of iron-rich foods, only 33% could name five iron-rich foods. More importantly, 12% of mothers could not name a single food rich in iron. This highlights the inadequacies in the mechanisms that transfer health-related messages to the public, especially for parents of preschool children. The most important document that provides health information regarding children in Sri Lanka is the Child Health Developmental Record (CHDR), issued to all children at birth [34]. Although it contains sufficient information on complementary feeding, it does not provide information on iron-rich foods which are freely available. Overall, the results of our study demonstrate major challenges for the healthcare system in Sri Lanka, including paediatricians, doctors, nurses and other healthcare workers.

Another important observation of the study is that maternal knowledge of anaemia had positive associations with the higher education level and increasing age of the mother. The association between better knowledge and higher education level is not surprising, and the association with the increasing age could be attributed to the greater experience of mothers regarding childcare. The results were comparable to the recent report from Ethiopia where the knowledge of anaemia was associated with a higher education level in women [24]. Father’s occupation status and the monthly family income did not show any associations with the knowledge on maternal knowledge of anaemia in our study.

One important strength of our study is that it is possibly the most comprehensive study that evaluates maternal knowledge of childhood anaemia in Sri Lanka. However, the study has a few limitations. Most importantly, the study did not evaluate the associations between the presence of anaemia in children with maternal knowledge. Considering the high prevalence of anaemia in Sri Lanka, it is important to accurately describe the country-specific risk factors for anaemia in children in future studies.

## Conclusions

In conclusion, this study revealed that the knowledge of anaemia and awareness of thalassaemia among mothers of preschool children is very poor. A very low proportion of mothers were aware of iron-rich food and feed it to their children. Despite being in a thalassaemia-endemic region, a very low proportion of mothers were aware that thalassaemia is a cause of anaemia and have got themselves screened for thalassaemia. Overall, it highlights the importance of intensifying the health promotion and thalassaemia prevention programmes of Sri Lanka to minimise the burden of childhood anaemia.

## Supplementary Information

**Additional file 1.** Data set.

## Data Availability

The data set supporting the conclusions of this article is included within the article and its additional files (Additional file 1).

## Abbreviations

AOR: Adjusted odds ratio
CHDR: Child Health Developmental Record
MOH: Medical Officer of Health
